# A circular network of adenosine-mediated mitochondrial dysfunction as coregulators of acute myocardial infarction

**DOI:** 10.7150/ijms.97066

**Published:** 2024-05-19

**Authors:** Yang Liu, Tianci Xiao, Zili Wang, Yangbin Ou, Ying Tan, Liting Chen, Na Zhou, Rongjun Zou

**Affiliations:** 1 The Traditional Chinese Medicine Department, Zhongshan Huangpu People's Hospital, Zhongshan,528429, Guangdong, China.; 2 School of Medicine, Southern University of Science and Technology (SUSTech), Shenzhen, Guangdong, China.; 3 Heart Center, Guangdong Provincial Key Laboratory of Research in Structural Birth Defect Disease, Guangzhou Women and Children's Medical Center, Guangzhou Medical University, Guangzhou 510623, China.; 4 The Second Clinical College of Guangzhou University of Chinese Medicine, Guangzhou 510405, Guangdong, China.; 5 Department of Cardiovascular Surgery, Guangdong Provincial Hospital of Chinese Medicine, the Second Affiliated Hospital of Guangzhou University of Chinese Medicine, Guangzhou 510120, Guangdong, China.

**Keywords:** Myocardial infarction, Molecular mechanisms, Mendelian Randomization analysis, Metabolomic mass spectrometry analysis, Transcriptome analysis, Gene-metabolite interaction network analysis

## Abstract

This study aims to explore the molecular mechanisms and associated pathways of myocardial infarction (MI). We employed a variety of analytical methods, including Mendelian Randomization (MR) analysis, transcriptome microarray data analysis, gene function and pathway enrichment analysis, untargeted metabolomic mass spectrometry analysis, and gene-metabolite interaction network analysis. The MR analysis results revealed a significant impact of mitochondrial DNA copy number on MI and coronary artery bypass grafting. Transcriptome analysis unveiled numerous differentially expressed genes associated with myocardial ischemia, with enrichment observed in cardiac function and energy metabolism pathways. Metabolomic analysis indicated a significant downregulation of mitochondrial regulation pathways in ischemic myocardium. T500 metabolite quantification analysis identified 90 differential metabolites between MI and Sham groups, emphasizing changes in metabolites associated with energy metabolism. Gene-metabolite interaction network analysis revealed the significant roles of key regulatory molecules such as HIF1A, adenosine, TBK1, ATP, NRAS, and EIF2AK3, in the pathogenesis of myocardial ischemia. In summary, this study provides important insights into the molecular mechanisms of MI and highlights interactions at multiple molecular levels, contributing to the establishment of new theoretical foundations for the diagnosis and treatment of MI.

## 1. Introduction

Myocardial infarction (MI) is a condition characterized by myocardial ischemia and necrosis due to reduced coronary artery blood flow, typically precipitated by thrombus formation 1, 2. According to a report by the World Health Organization (WHO), approximately 18 million people worldwide died from cardiovascular diseases (CVDs) in 2019, representing 32% of the total global mortality and emerging as a leading cause of death among all diseases. Within the realm of cardiovascular diseases, MI resulting from coronary heart disease accounts for roughly 75% of cases of sudden cardiac death (SCD), thus presenting a significant international health challenge 1, 2. Despite advancements in treatments such as anticoagulants, antiplatelet agents, thrombolysis, and reperfusion therapy, the incidence of MI remains notably high. However, some patients still progress to heart failure, underscoring the urgent need for effective therapeutic strategies to alleviate myocardial damage. The pathophysiological mechanisms of MI involve the development of atherosclerosis, encompassing a complex array of biological processes including low-density lipoprotein (LDL) oxidation, endothelial cell injury, inflammatory responses, and thrombus formation 3, 4. The primary pathological feature of MI is diminished coronary artery blood flow leading to myocardial ischemia and necrosis, typically triggered by thrombus formation. Apart from thrombus formation, other mechanisms such as vasospasm and coronary artery anatomical abnormalities may also contribute to MI. Treatment modalities for MI encompass pharmacological interventions such as anticoagulants, antiplatelets, thrombolysis, and reperfusion therapy, alongside interventional procedures including percutaneous coronary intervention (PCI) and coronary artery bypass grafting (CABG) 2. In recent years, nanomaterials have emerged as a novel therapeutic approach, showcasing immense potential. Nanomaterials can serve as drug delivery systems, reducing drug dosage and frequency, mitigating side effects, and enhancing efficacy. Additionally, nanomaterials can promote local vascular regeneration, alleviate myocardial damage, improve local mitochondrial-related metabolic remodeling, and potentially chart new directions for the future treatment of MI 4. However, current MI treatments still encounter limitations. For instance, some patients, despite receiving state-of-the-art treatments, still progress to heart failure, highlighting the constraints of existing therapeutic methods. Furthermore, while nanomaterials exhibit potential in MI treatment, their clinical application remains in its nascent stages, necessitating further research and clinical trials to validate their safety and efficacy.

The occurrence and progression of MI and heart failure are influenced by various factors, including mitochondrial dysfunction. Mitochondria serve as the energy centers of cardiac cells, maintaining their functionality and quantity through fusion and fission processes 3. Recent research indicates that mitochondrial fusion and fission play pivotal roles in the occurrence and progression of MI and heart failure, emerging as a focal point of investigation. During the onset and progression of MI, mitochondrial fusion serves as a crucial mechanism for sustaining cardiac cell survival and function 3. When MI occurs, cardiac cells undergo ischemic and reperfusion injuries, leading to a significant increase in oxidative stress levels. Oxidative stress damages mitochondrial function, thereby affecting cardiac cell viability 5. In such circumstances, mitochondrial fusion aids in preserving mitochondrial functional integrity, mitigating oxidative stress-induced mitochondrial damage, and consequently protecting cardiac cells from injury. Additionally, abnormal proliferation and fission of mitochondria are key features during various forms of cell death such as apoptosis and necrosis 6. Mitochondrial fission leads to an increase in intracellular mitochondrial quantity, thereby exacerbating oxidative stress and cell death processes. During MI, cardiac oxidative stress is a crucial pathophysiological mechanism directly impacting mitochondrial function and structure 5, 7. Elevated levels of oxidative stress can impair mitochondrial function, including loss of mitochondrial membrane potential, decreased oxidative phosphorylation capacity, and excessive reactive oxygen species (ROS) production, thereby triggering mitochondrial fission.

Mitochondrial fusion/fission processes help maintain mitochondrial quantity and functionality, facilitating the restoration of energy metabolism and protecting cardiac cells from the effects of energy insufficiency. Furthermore, during MI occurrence, certain cardiac cells may suffer severe ischemic and reperfusion injuries, resulting in apoptosis and necrosis. Mitochondrial fusion promotes the survival of damaged cardiac cells, reducing the number of apoptotic cells and maintaining the integrity and functionality of cardiac tissue 5, 7. Additionally, the activation of inflammatory cells and release of inflammatory mediators further promote mitochondrial fission and abnormal proliferation. Concurrently, mitochondrial fission may exacerbate the severity of MI by promoting the escalation of inflammatory responses. Recent studies suggest that mitochondrial fusion/fission processes and dynamics anomalies may also participate in angiogenesis, facilitating the repair and regeneration of damaged cardiac tissue. By modulating mitochondrial homeostasis, it is possible to enhance the survival and regenerative capacity of cardiac cells, thereby aiding in the restoration of cardiac function 6, 8.

Therefore, investigating mitochondrial metabolism is of paramount importance for delving into the mechanisms and clinical applicability of MI. A thorough comprehension of mitochondrial metabolic pathways facilitates the elucidation of the pathophysiological underpinnings of MI, clarifies the interplay between oxidative stress and mitochondrial impairment, and probes into the regulatory mechanisms governing mitochondrial dynamics.

## 2. Methods

### 2.1 The Mendelian Randomization (MR) analysis

The MR analysis conducted in this study utilized five datasets sourced from publicly available GWAS summary data (https://gwas.mrcieu.ac.uk/) 9, 10. The mitochondrial DNA copy number data was derived from a meta-analysis study comprising 383,476 samples of European ancestry (ID: EBI-A-GCST90026372), while the summary data for coronary artery bypass grafting included 547,261 samples of European ancestry, and myocardial infarction data originated from a meta-analysis study encompassing 462,933 samples of European ancestry. Data analysis was performed using R (version 4.2.1) with the TwoSampleMR (0.5.6) and MRPRESSO (1.0) packages 9. The MR analysis primarily employed the Inverse Variance Weighted (IVW) method, which utilizes the inverse of the variance of each genetic variant's effect size to weight the effects. Additionally, four other statistical methods, namely the Weighted Median Estimator (WME), Weighted Model-Based Method (WM), MR-Egger Regression (MER), and Simple mode (SE), were applied to assess the association between genetic variants and diseases, estimate causal effects, and evaluate bias and symmetry of the estimation values, as well as the stability and consistency of the results 9. Harmonization was employed to remove SNPs with incompatible alleles and those with allele frequencies being palindromic. Given potential heterogeneity in SNP extraction across different experimental settings, leading to heterogeneity in two-sample MR analysis and subsequent errors in causal inference, heterogeneity testing and MR-Egger regression were conducted on the main IVW analysis method to address this concern. Regarding horizontal pleiotropy in MR analysis, intercept values from MR-Egger were utilized to evaluate its presence, with P-values from the heterogeneity test indicating its significance 10. A P-value above 0.05 suggested negligible horizontal pleiotropy in the causal analysis. Finally, leave-one-out analysis was employed to assess result consistency.

### 2.2 Transcriptome microarray data analysis

DESeq2 is a widely used tool for analyzing RNA-seq data, primarily employed to detect differential gene expression under different conditions 11. It is based on a negative binomial generalized linear model, which statistically evaluates the number of sequence fragments for each gene, enabling comparison of gene expression levels across different conditions. DESeq2 features data-driven prior distributions to estimate dispersion and logarithmic fold changes, thereby enhancing the accuracy and reliability of the analysis 12, 13. The Count expression value data for each gene in each sample was downloaded from the Gene Expression Omnibus (GEO; https://www.ncbi.nlm.nih.gov/geo/) database 14, specifically from GSE110209 15. Using the DESeqDataSetFromMatrix function, the Count expression value data, sample information, and experimental design information were integrated into a DESeqDataSet object. DESeq2 performs data normalization, batch effect removal, and other data cleaning and correction operations on the dataset to ensure the accuracy of the analysis. The DESeq() function was applied to conduct differential analysis on the DESeqDataSet object. This function calculates the differential expression levels for each gene and performs statistical significance tests on the differential expression, resulting in a list of differentially expressed genes along with associated statistical information 12, 13. Differential genes were identified based on an FDR-adjusted p-value less than 0.05 and an absolute log fold change greater than or equal to 1.0.

### 2.3 Gene functional and pathway enrichment analysis

Here, both Gene Ontology (GO) and Kyoto Encyclopedia of Genes and Genomes (KEGG) analyses were conducted to elucidate the functional and pathway enrichment of genes. The GO analysis classified genes into three main groups: biological processes (BP), cellular components (CC), and molecular functions (MF) 16. The clusterProfiler, enrichKEGG, enrichplot, and enrichGO methods were employed for this analysis 16, where terms with adjusted p-values less than or equal to 0.05, determined using the Benjamini & Hochberg method, were considered significantly enriched. Additionally, for pathway visualization, barplot and cnetplot functions from the enrichplot package were utilized to generate a bar chart and a category net plot, respectively.

Moreover, Gene Set Enrichment Analysis (GSEA) and Gene Set Variation Analysis (GSVA) were performed using gene sets obtained from the Molecular Signatures Database (MsigDB) 17, 18. Gene sets were downloaded in gmt format and imported into R using the getGmt function in the GSEABase package. For GSEA, clusterProfiler, gseGO, and gseKEGG functions were employed, utilizing a ranked list of genes, species identifier, and 1000 permutations for the enrichment test. On the other hand, GSVA analysis was conducted using the gsva function, applied to the expression data matrix, gene set collection, and specified calculation method, resulting in GSVA scores for each sample 17, 18. Subsequently, these scores were utilized for downstream analyses, including differential expression analysis based on the LIMMA method.

### 2.4 Untargeted Metabolomic Mass Spectrometry Analysis and Quantitative Analysis with T500

Specifically, we selected C57BL/6N mice aged 8-10 weeks and weighing between 20-25g as research subjects and induced myocardial infarction (MI) by ligating the left anterior descending artery (LAD) under anesthesia. All animal care and experimental procedures were approved by Committee on Animal Research and Ethics of Guangzhou Medical University (Acceptance number: G2020-073). Following tissue sample collection and preparation, LC-MS technology was employed for mass spectrometry data collection and analysis. And this study leverages the novel metabolomic profiling technology provided by Wuhan Metware Biotechnology Co., Ltd. (https://www.metware.cn), which combines the advantages of untargeted "comprehensive" and targeted "accurate" metabolomic approaches, offering high throughput, ultra-sensitivity, broad coverage, and accurate qualitative and quantitative capabilities. This technology utilizes chromatography-mass spectrometry coupling to accomplish the entire process from substance separation to identification, with liquid chromatography-tandem mass spectrometry (LC-MS/MS) enabling precise quantification 19. Metabolomic analysis aims to detect and screen metabolites of significant biological and statistical differences in biological samples, revealing insights into metabolic processes and mechanisms of change. The analysis comprises two main components: experimental procedures and data analysis, involving differential metabolite screening and metabolic pathway analysis 20. Through this technology, over 3000 metabolites can be simultaneously qualitatively and quantitatively detected in biological samples. In the experiments, both hydrophilic and hydrophobic substance extraction methods were utilized, and data acquisition was conducted using a chromatography-mass spectrometry system, including ultra-high-performance liquid chromatography and tandem mass spectrometry 21. Metabolite qualitative analysis relied on a self-constructed targeted standard product database, MWDB, utilizing retention time and precursor and fragment ions for metabolite identification. Quantitative analysis depended on multiple reaction monitoring (MRM) mode of triple quadrupole mass spectrometry (MS), enabling precise and reproducible quantification 20. The entire analysis process involves sample preparation, data acquisition and analysis, and result interpretation, providing important technical support and data foundation for metabolomic research.

In data processing, chromatographic peaks were aligned, and normalization and appropriate missing value imputation methods were applied. For statistical analysis, orthogonal partial least squares discriminant analysis (OPLS-DA) was conducted, allowing the identification of significant metabolites distinguishing control and MI groups. Differential expressed metabolites (DEMs) screening was based on two key criteria: VIP scores ≥ 1 and fold changes ≥ 2 or ≤ 0.5 between control and experimental groups. Metabolite interactions form various pathways, annotated using the Kyoto Encyclopedia of Genes and Genomes (KEGG) database. Subsequently, for quantitative analysis of core metabolites, we employed a novel technology product called T500 (also procured from Wuhan Metware Biotechnology Co., Ltd.; https://www.metware.cn). T500 is an absolute quantitative detection analysis technology for metabolomic mass spectrometry, covering 540 target substances related to energy metabolism, tryptophan pathway, fatty acids, bile acids, neurotransmitters, steroid hormones, amino acids, organic acids, and trimethylamine oxidation. This technology enables simultaneous qualitative and quantitative analysis across nine major metabolic pathways, providing a scientific tool and method for studying the occurrence and progression of diseases.

### 2.5 Gene-Metabolite Interaction Network Detection

Using the Network Analysis module in MetaboAnalyst (version 6.0; https://www.metaboanalyst.ca/) 22, we constructed a Gene-Metabolite Interaction Network by inputting core genes and differentially expressed metabolites. This network analysis tool enables researchers to explore and visualize interactions between relevant metabolites and genes. We extracted associations between chemical substances and human genes from the STITCH database, focusing on highly credible interactions. These associations are primarily based on co-mentions highlighted in PubMed abstracts, indicating similarities in chemical structure and molecular activity. Additionally, based on the results obtained from MetaboAnalyst online analysis, we further conducted network analysis using Cytoscape software. Utilizing the cytoHubba plugin in Cytoscape 23, we analyzed core regulatory subgroups within the overall network. By employing the Maximum Clique Centrality (MCC) algorithm, we iteratively narrowed down the core subset of genes and metabolites, ultimately identifying the most critical gene-metabolite regulatory relationships.

## 3. Results

### 3.1 MR Analysis Results

**Figure 1:**
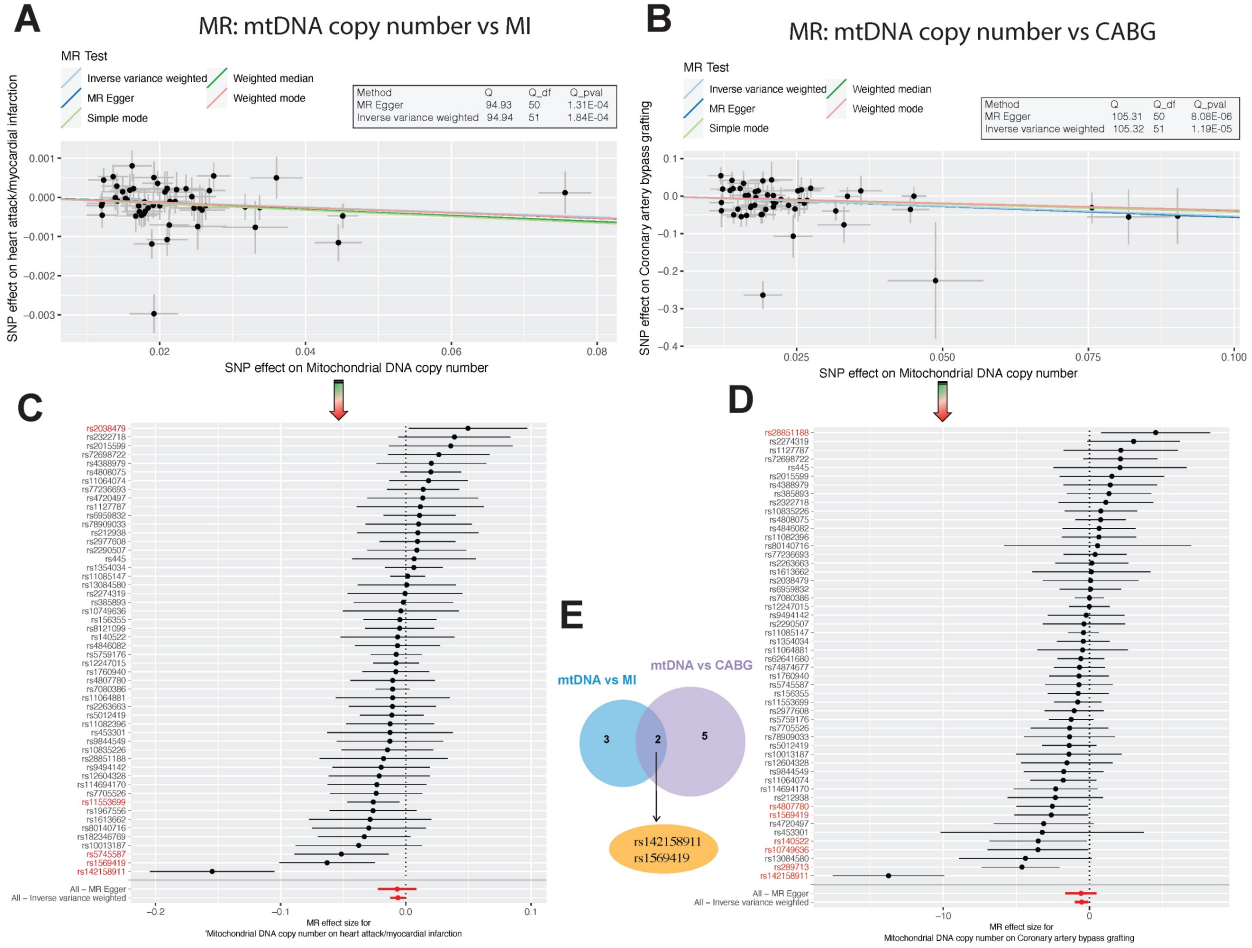
** Association Analysis of Mitochondrial DNA Copy Number with Myocardial Infarction and Coronary Artery Bypass Grafting.** (A-B). Scatter plots illustrating the allelic expression magnitudes between Mitochondrial DNA Copy Number (mtDNA CN) and Myocardial Infarction, as well as Coronary Artery Bypass Grafting (CABG). (C-E). Schematic representation of differential Single Nucleotide Polymorphism (SNP) loci: There are 5 differential loci between mtDNA CN and Myocardial Infarction, 7 between mtDNA CN and CABG, with 2 core differential loci shared between both conditions, namely rs142158911 and rs1569419.

In our MR analysis, we initially selected 67 SNPs as instrumental variables (IVs) using Mitochondrial DNA copy number as the exposure factor, with myocardial infarction as the outcome factor. The results of the Inverse Variance Weighted (IVW) analysis revealed an odds ratio (OR) of 0.99372 (95% CI=0.98785-0.99961, P=0.03) for myocardial infarction. Similarly, using Coronary artery bypass grafting as the exposure factor and extracting 75 SNPs, the IVW analysis showed an OR of 0.97672 (95% CI=0.95954-0.99421, P=0.009) for Mitochondrial DNA copy number as the outcome factor (Table 1). These findings indicate a significant impact of Mitochondrial DNA copy number on myocardial infarction and Coronary artery bypass grafting (**Figure 1C-D**). Furthermore, Cochran's Q analysis revealed heterogeneity between Mitochondrial DNA copy number and myocardial infarction, as well as Coronary artery bypass grafting (P<0.05) (Table 2). This heterogeneity may arise from fixed SNP loci, potentially affecting sample selection in dual-sample settings. However, this does not undermine the reliability of the IVW method's conclusions. Moreover, MR-Egger analysis indicated no horizontal pleiotropy between Mitochondrial DNA copy number and myocardial infarction, as well as Coronary artery bypass grafting (Table 3). Scatter plots depicting SNP expression magnitudes between Mitochondrial DNA copy number and myocardial infarction, and Coronary artery bypass grafting, were illustrated in **Figure 1A-B**. To estimate the strength of the instrumental variables, we calculated R² and the f-statistic using the allele frequency (EAF) and effect estimate (BETA) in the presence of an effective allele frequency value. All f-statistic values exceeded 10. Notably, there were 5 differential SNP loci between Mitochondrial DNA copy number and myocardial infarction, 7 between Mitochondrial DNA copy number and Coronary artery bypass grafting, and 2 shared core differential loci, namely rs142158911 and rs1569419 (**Figure 1E**).

### 3.2 Transcriptomic Microarray Analysis of Myocardial Infarction in Mice

**Figure 2:**
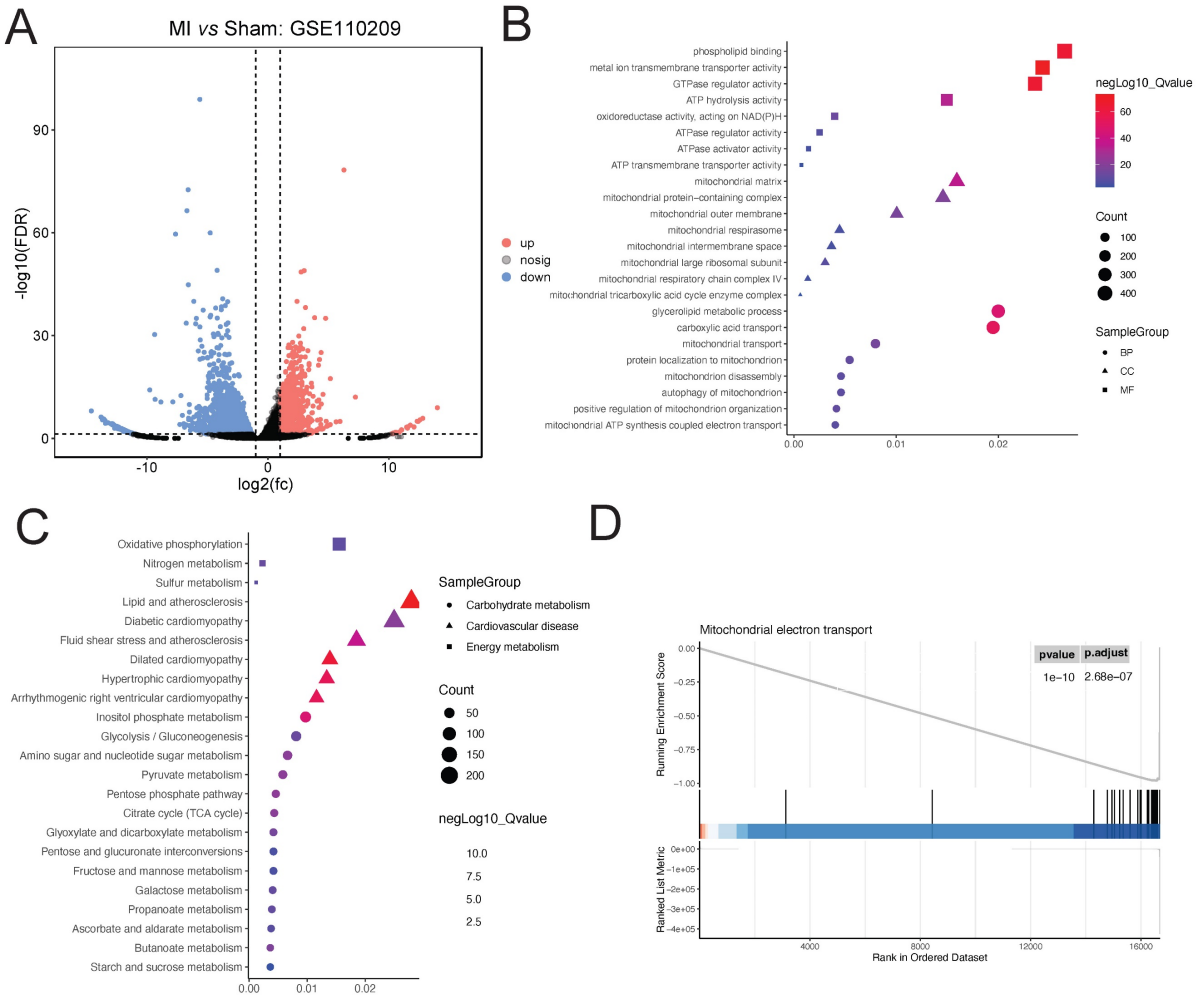
** Analysis of Ischemic Cardiac Tissue Reveals Dysregulated Pathways.** (A). DESeq2 analysis reveals 2255 upregulated and 895 downregulated genes compared to Sham controls. (B). GO enrichment highlights significant biological processes, cellular components, and molecular functions. (C). KEGG pathway analysis indicates enrichment of cardiac and lipid metabolism pathways. (D). GSEA identifies significant enrichment of the Mitochondrial electron transport pathway.

Downloaded GSE110209 count data from GEO was analyzed for differential expression using DESeq2. A total of 2255 upregulated and 895 downregulated genes were identified in the myocardial ischemia group compared to the Sham group (**Figure 2A**). GO enrichment analysis of the differentially expressed genes revealed significant enrichment in several BP terms including phospholipid binding, metal ion transmembrane transporter activity, and GTPase regulator activity. Additionally, terms related to CC such as mitochondrial matrix, mitochondrial protein-containing complex, and mitochondrial outer membrane were significantly enriched. Furthermore, MF terms like glycerolipid metabolic process, carboxylic acid transport, and mitochondrial transport showed significant enrichment (**Figure 2B**). Moreover, KEGG enrichment analysis revealed significant enrichment in pathways associated with Dilated cardiomyopathy, Hypertrophic cardiomyopathy, Arrhythmogenic right ventricular cardiomyopathy, Lipid and atherosclerosis, and Inositol phosphate metabolism, as well as relevant energy metabolism pathways (**Figure 2C**). Furthermore, GSEA indicated significant enrichment of the Mitochondrial electron transport pathway (ES=0.94; NES=-1.64; adjusted P-value=2.68e-07) (**Figure 2D**).

### 3.3 Untargeted Metabolomic Mass Spectrometry Analysis

**Figure 3:**
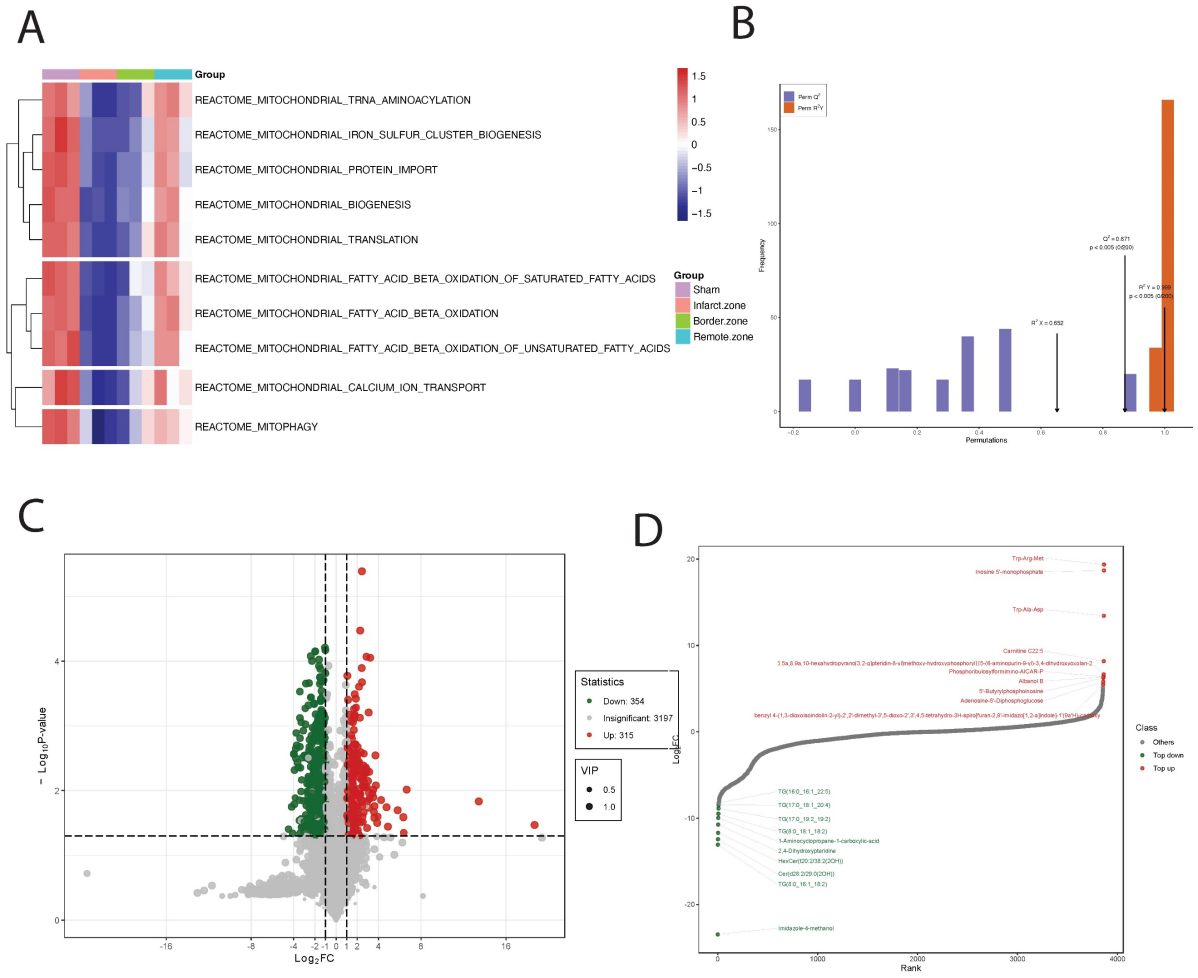
** Analysis of Mitochondrial Regulation and Metabolomic Profiling in Ischemic Cardiac Tissue**. (A). GSVA analysis reveals reduced mitochondrial pathway enrichment scores in ischemic myocardium compared to Sham controls, with normalization in the distal region. (B). OPLS-DA-based metabolomic profiling establishes a highly predictive model with strong explanatory and predictive capabilities. (C). Analysis identifies 739 differential metabolites (DEMs), including both upregulated and downregulated species. (D). Specific metabolites, such as Trp-Arg-Met and Imidazole-4-methanol, show significant dysregulation.

**Figure 4:**
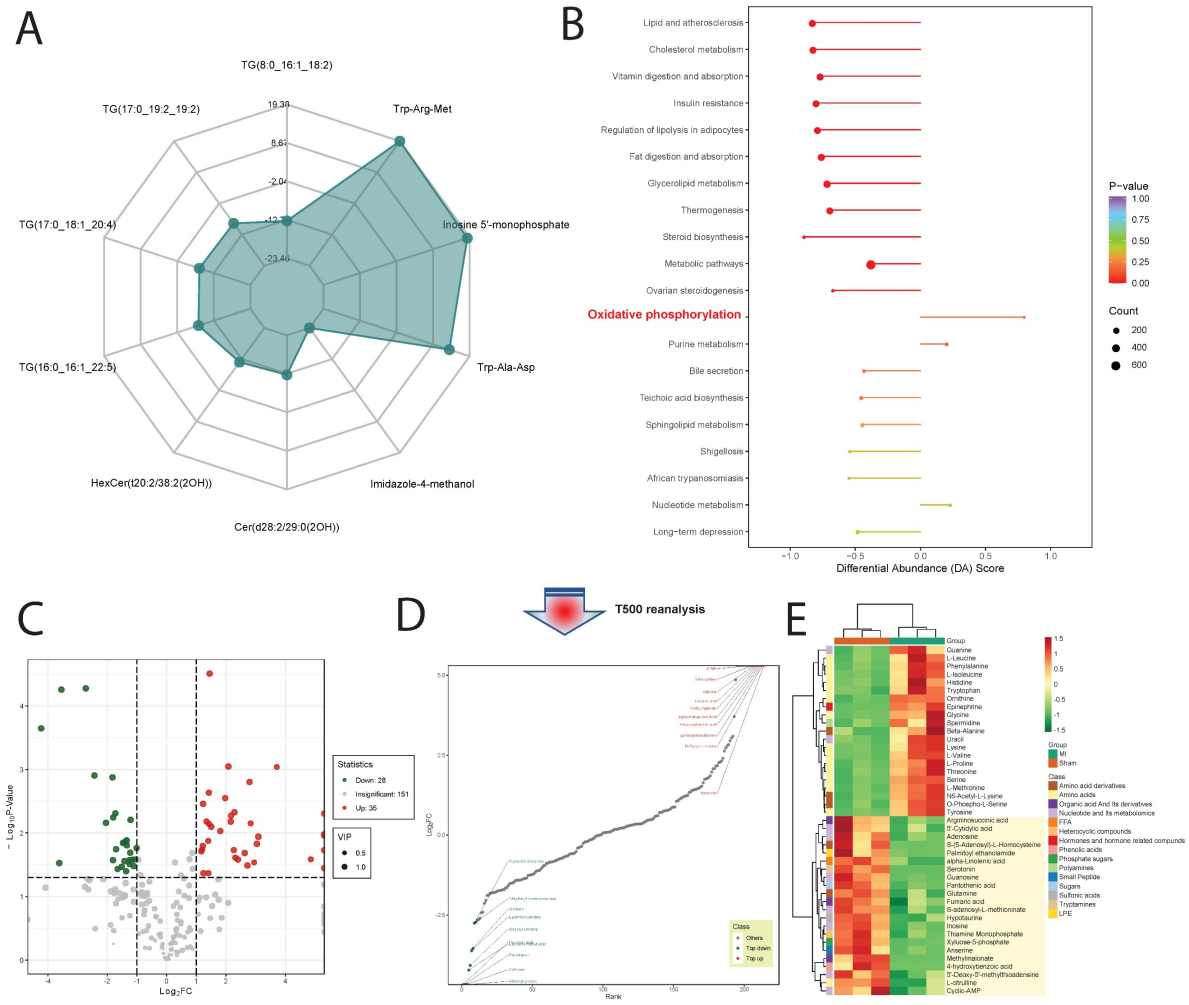
** Metabolite Profiling and Pathway Enrichment in Ischemic Cardiac Tissue**. (A). Radar chart shows significant upregulation of metabolites like Trp-Arg-Met and downregulation of others such as Imidazole-4-methanol. (B). Pathway enrichment analysis reveals significant scores for Oxidative phosphorylation, Steroid biosynthesis, and Cholesterol metabolism pathways. (C). Using T500 quantitative metabolite detection technology, we identified 90 differential metabolites between MI and Sham groups, with 44 downregulated and 46 upregulated DEMs. (D). Specific metabolites like Serotonin and L-Proline are significantly upregulated, while Glycine and L-Valine are significantly downregulated. (E). Row-wise normalization of raw abundance of differential metabolites highlights significant differences in energy metabolism-related metabolites.

Upon GSVA analysis of the GSE110209 dataset, we observed significant decreases in pathway enrichment scores related to mitochondrial regulation in the myocardial ischemia group compared to the Sham group. Specifically, pathways such as REACTOME: mitochondrial TRNA aminoacylation, REACTOME: mitochondrial iron sulfur cluster biogenesis, and REACTOME: mitochondrial protein import exhibited markedly reduced scores in the ischemic myocardium. Conversely, in the ischemic border zone, these scores showed a decreasing trend but remained significantly lower. Notably, in the ischemic distal region (relative to normal tissue), the scores for mitochondrial-related regulatory pathways were significantly elevated and comparable to those of the Sham group (**Figure 3A**). Through comprehensive metabolomic profiling using OPLS-DA, we established a robust predictive model with satisfactory parameters. The model exhibited high explanatory power for both X and Y matrices, as indicated by R^2^X=0.652 and R^2^Y=0.999 (p < 0.05), respectively. Moreover, the model demonstrated strong predictive capability, with a Q^2^ value of 0.872, signifying an effective and reliable model approaching excellence (**Figure 3B**). Subsequently, we identified a total of 739 DEMs, comprising 130 downregulated and 609 upregulated DEMs (Figure 3C). Notably, certain metabolites such as Trp-Arg-Met, Inosine 5'-monophosphate, and Trp-Ala-Asp showed significant upregulation, while others including Imidazole-4-methanol, TG(8:0_16:1_18:2), and Cer(d28:2/29:0(2OH)) exhibited significant downregulation (**Figure 3D and Figure 4A**). Upon pathway enrichment analysis of the DEMs, we observed that Oxidative phosphorylation, Steroid biosynthesis, and Cholesterol metabolism exhibited the most significant Differential Abundance Scores (**Figure 4B**).

### 3.4 T500 Metabolite Quantification Analysis

Using T500 quantitative metabolite detection technology, we identified 90 differential metabolites in myocardial tissue between the MI and Sham groups, with 44 downregulated DEMs and 46 upregulated DEMs (**Figure 4C**). Among these, Serotonin, 12-Hydroxyoleic acid, and N-Glycyl-L-Leucine were significantly upregulated DEMs, while L-Proline, Glycine, and L-Valine were significantly downregulated DEMs (**Figure 4D**). To facilitate the observation of changes in metabolite levels, we normalized the raw abundance of differential metabolites identified using the applied selection criteria by row-wise normalization (Unit Variance Scaling, UV Scaling). We found that energy metabolism-related metabolites were significantly different DEMs (**Figure 4E**).

**Figure 5:**
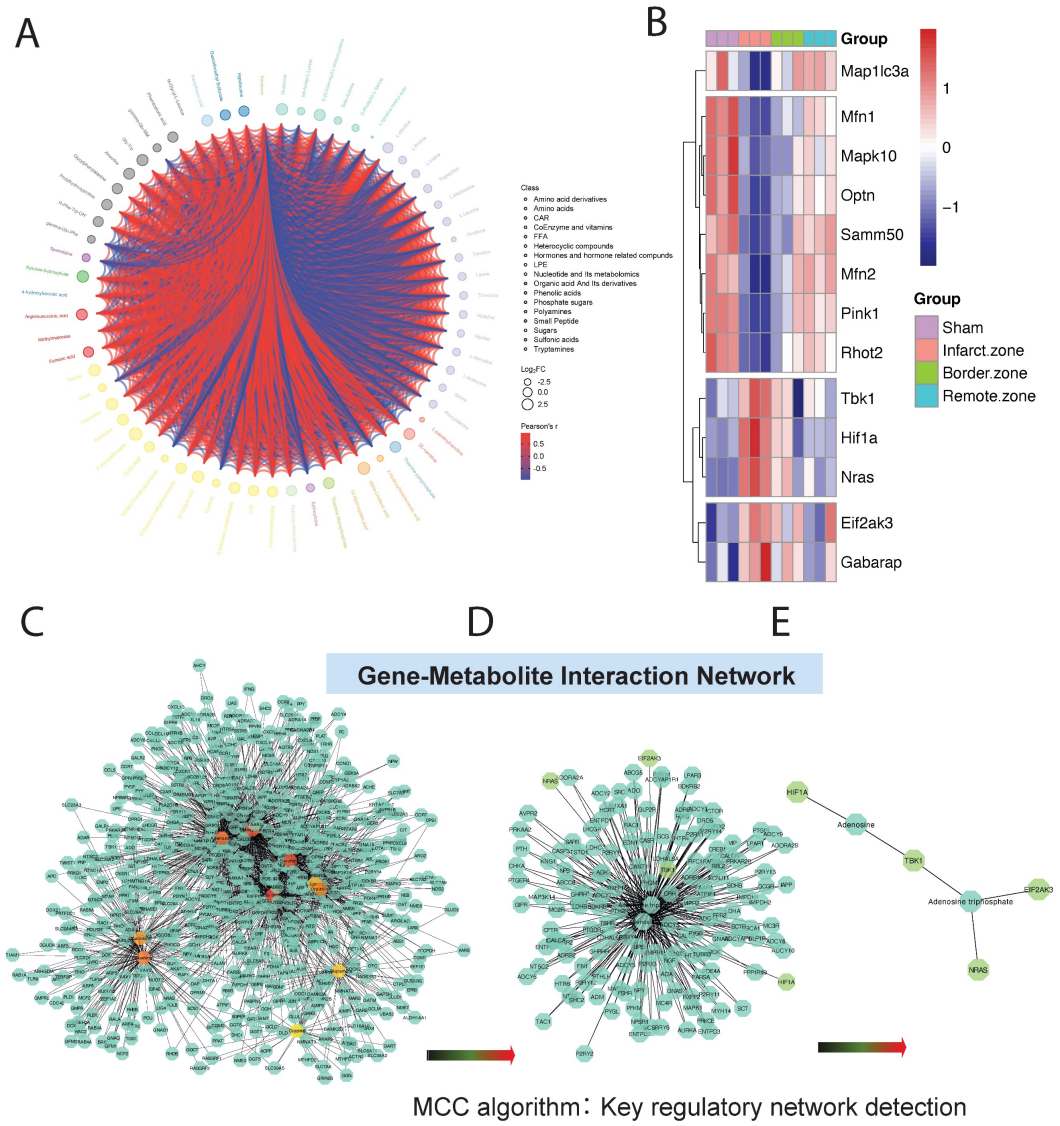
** Metabolic and Gene Expression Analysis in Ischemic Cardiac Tissue.** (A).Pearson correlation analysis reveals associations between metabolites, particularly energy metabolism-related DEMs. (B). Heatmap displays expression patterns of core genes from GSEA mitochondrial enrichment analysis. Genes like Map1lc3a and Mfn1 show decreased expression in ischemic tissue but increase in ischemic border and distal regions, while TBK1 and HIF1A exhibit opposite trends. (C-E). Interaction regulatory network between myocardial regulatory genes and DEMs quantified by T500, visualized using Cytoscape. Core regulatory molecules include HIF1A, Adenosine, TBK1, Adenosine triphosphate, NRAS, and EIF2AK3.

DEMs may exhibit synergistic or antagonistic relationships, and correlation analysis can help assess the metabolic proximity between significantly different metabolites, aiding in further understanding the regulatory relationships between metabolites during changes in biological states. Pearson correlation analysis was performed on differentially significant metabolites identified based on selection criteria, revealing close associations between various classes of metabolites, particularly energy metabolism-related DEMs (**Figure 5A**).

### 3.5 Gene-Metabolite Interaction Network Detection

To further investigate the mitochondrial regulatory mechanisms, we conducted differential expression analysis on core genes obtained from GSEA mitochondrial enrichment and visualized them using a heatmap. Among these genes, Map1lc3a, Mfn1, Mapk10, Optn, Samm50, Mfn2, Pink1, and Rhot2 exhibited significantly decreased expression in myocardial ischemic tissue compared to normal tissue, while their expression gradually increased in ischemic border and distal regions relative to normal tissue. Conversely, TBK1, HIF1A, NRAS, EIF2AK3, and GABARAP showed opposite expression trends across different myocardial tissue groups (**Figure 5B**). Using MetaboAnalyst online tools, we constructed an interaction regulatory network between the above myocardial regulatory genes and DEMs quantified by T500. The network was visualized using Cytoscape software, and the MCC algorithm in the cytoHubba plugin was employed to identify core regulatory subgroups within the interaction network (**Figure 5C-E**). Ultimately, we identified a core regulatory network composed of HIF1A, Adenosine, TBK1, Adenosine triphosphate, NRAS, and EIF2AK3 as key regulatory molecules.

## 4. Discussion

A comprehensive analysis of the metabolic and gene expression profiles in acute ischemic cardiac tissue has provided profound insights into the molecular mechanisms underlying MI. Our research findings have unveiled dysregulated metabolites and genes intricately linked to mitochondrial function, energy metabolism, and cellular homeostasis, thereby holding significant implications within the context of myocardial ischemia. Metabolomic analysis has delineated substantial alterations in metabolite abundance in ischemic tissue compared to controls, with select metabolites exhibiting marked upregulation or downregulation. Notably, metabolites associated with energy metabolism pathways have displayed pronounced dysregulation, suggesting perturbation of cellular energy metabolism in response to ischemic insult. Radar plot visualization has underscored notable changes in specific metabolites, such as Trp-Arg-Met and Imidazole-4-methanol, offering potential biomarkers for diagnostic and therapeutic targeting of MI. Pathway enrichment analysis of differential metabolites has unveiled involvement of key metabolic pathways, including oxidative phosphorylation, steroid biosynthesis, and cholesterol metabolism, which undergo substantial alterations in ischemic cardiac tissue. These pathways play pivotal roles in cellular energy production, lipid metabolism, and cell membrane structure, implicating their dysregulation in the pathogenesis of ischemic heart disease. Additionally, correlation analysis has unveiled intricate associations among different metabolite categories, particularly those pertinent to energy metabolism, emphasizing the intricate nature of metabolic regulation in ischemic cardiac tissue. In terms of gene expression, differential expression analysis has revealed alterations in core genes associated with mitochondrial function and cellular stress response. Genes such as Map1lc3a and Mfn1 have exhibited diminished expression in ischemic tissue yet augmented expression in the ischemic border and remote regions, indicating dynamic alterations in mitochondrial dynamics and autophagy in response to ischemic insult. Conversely, genes such as TBK1 and HIF1A have demonstrated contrasting expression trends, reflecting the diverse regulatory mechanisms underlying MI pathophysiology.

Ultimately, we have identified a cohort of core regulatory molecules, including HIF1A, adenosine, TBK1, ATP, NRAS, and EIF2AK3, which play pivotal roles in the pathogenesis of MI. These regulatory molecules intricately interact to form a complex regulatory network involving cellular energy metabolism, autophagy, inflammatory response, and stress response.

Hypoxia-inducible factor-1α (HIF-1α) assumes a crucial role in MI. Characterized by ischemic necrosis of myocardial tissue consequent to coronary artery occlusion, MI precipitates severe cardiac damage and inflammatory response 24. Under hypoxic conditions, HIF-1α expression significantly escalates, likely facilitating cellular adaptation to hypoxia. Activation of HIF-1α can modulate the development and outcomes of MI through multifaceted pathways. Primarily, HIF-1α activation fosters angiogenesis and sustenance of vascular function. During myocardial infarction, ischemic myocardial tissue necessitates heightened vascularization for oxygen and nutrient provision 25, 26. HIF-1α orchestrates angiogenesis and vasodilation by modulating the expression of vascular endothelial growth factor (VEGF) and other genes, thereby ameliorating blood supply to ischemic myocardium. Secondly, HIF-1α activation also impacts myocardial cell metabolism. In myocardial infarction, metabolic activity of myocardial cells is disrupted due to hypoxia and energy metabolism perturbations. HIF-1α promotes activation of the glycolytic pathway, bolstering lactate production and ATP synthesis to sustain myocardial cell viability. Furthermore, HIF-1α participates in regulating myocardial cell survival and apoptosis. Throughout myocardial infarction, hypoxia and reactive oxygen species production may induce apoptosis and necrosis of myocardial cells. Activation of HIF-1α shields myocardial cells from hypoxia-induced damage by modulating the expression of apoptosis-related genes, such as members of the Bcl-2 family and apoptosis regulatory factors 25, 26. Adenosine emerges as a pivotal cellular signaling molecule released under ischemic conditions, regulating cellular metabolism and safeguarding cells from hypoxic and oxidative stress damage. A close association exists between myocardial infarction and adenosine. Released during cellular or tissue hypoxia or ischemia, adenosine, alongside ATP and ADP, instigates reactive hyperemia. Reactive hyperemia denotes transient augmentation in blood flow ensuing re-opening of blood vessels after tissue blood supply cessation 27, 28. During myocardial infarction, tissue hypoxia and ischemia precipitate adenosine release from cells, which interacts with adenosine receptors on the cell surface, eliciting a gamut of physiological effects. Studies underscore the pivotal role of adenosine receptor A2AR in myocardial infarction. Activation of A2AR regulates coronary artery blood flow, fostering blood perfusion in myocardial tissue. Additionally, A1R negates the reactive hyperemia mediated by A2AR, further modulating myocardial perfusion 29. While controversy surrounds the role of adenosine in coronary reactive hyperemia, its role in myocardial infarction remains widely scrutinized and acknowledged. Nonetheless, certain studies have cast doubt on the precise role of adenosine in myocardial infarction, positing adenosine as not being a decisive factor in myocardial infarction development. In select animal models and clinical studies, a direct correlation between adenosine levels and myocardial infarction severity remains elusive 27, 28. TBK1 emerges as a multifaceted protein kinase modulating cellular autophagy processes and inflammatory response, thereby influencing cell viability and apoptosis. As a downstream kinase of the cGAS-STING signaling pathway, TBK1 assumes a pivotal role in the development of MI 30. MI entails ischemic necrosis of myocardial tissue, culminating in severe cardiac damage and inflammatory response. Studies indicate that abundant ischemic cell death and influx of cellular debris in the MI disease model trigger activation of the cGAS-STING-IRF3 signaling pathway. Activation of this pathway further exacerbates MI-associated inflammatory response, thereby exacerbating the dire consequences of MI. Furthermore, in heart failure, cGAS-STING signaling gradually escalates, further fostering pathological cardiac remodeling and left ventricular dysfunction 30-32. Adenosine triphosphate (ATP) stands as a pivotal intracellular energy molecule, adapting cellular energy demands amidst hypoxic conditions by modulating adenosine triphosphate-sensitive potassium channels (KATP channels) activity, thereby engaging in regulating cell electrical activity and function. KATP channels regulate their opening and closing states by modulating cell metabolic status, thereby shielding myocardial cells from metabolic stress-induced damage 33, 34. During myocardial infarction, ATP concentration diminishes, prompting KATP channel activation, thereby inhibiting cellular hyperexcitability and reducing energy consumption, thus alleviating myocardial injury. Furthermore, mutual interaction between ATP and KATP channels is regulated by intracellular factors such as magnesium ion concentration, ATP, and ADP concentrations. These factors collectively influence KATP channel activity, subsequently impacting myocardial cell function and survival 33, 34. NRAS participates in regulating multiple signaling pathways, including the MAPK signaling pathway, which play crucial roles in myocardial cells. During myocardial infarction, aberrant activation or mutations of NRAS might impede normal functioning of these signaling pathways, thus affecting myocardial cell survival and function 35. Lipid modifications of NRAS, such as saturation and prenylation, and other post-translational modifications, like phosphorylation, influence its localization on the cell membrane. This localization is critical for maintaining normal myocardial cell function, as the cell membrane serves as a key site for signal transduction. The Cys118 residue of NRAS might be susceptible to oxidative stress, potentially altering its stability, activity, and interactions, thereby influencing myocardial cell function and survival 35. Additionally, NRAS might play a role in oxidative stress through modifications such as S-nitrosylation. EIF2AK3 stands as an endoplasmic reticulum stress sensor, closely associated with endoplasmic reticulum protein quality control (ER-PQC) and unfolded protein response (UPR), protein kinase pivotal in myocardial cell growth and responses to physiological and pathological challenges 36. Under physiological conditions, such as exercise and pregnancy, myocardial cells undergo adaptive growth, resulting in myocardial hypertrophy, rather than pathological growth. This growth process is associated with increased protein synthesis, placing higher demands on endoplasmic reticulum protein folding mechanisms. However, the unfolded protein response components in the adult heart seem adequate to meet these demands, and thus, myocardial cell death is not observed under these conditions 35-37. Therefore, EIF2AK3 might modulate myocardial cell responses to the increased protein synthesis requisite for physiological growth, contributing to myocardial function preservation. Conversely, under pathological conditions such as myocardial infarction, myocardial cells endure severe damage, culminating in cell death and pathological changes in myocardial tissue. Under these circumstances, expression of UPR components often escalates, although whether this escalation is the cause or consequence of myocardial defects remains unclear. Thus, EIF2AK3 might influence myocardial cell responses to physiological and pathological conditions by modulating endoplasmic reticulum protein quality control and unfolded protein response, thereby affecting the development and progression of myocardial infarction 38, 39. Nonetheless, elucidating the precise role of EIF2AK3 in myocardial infarction warrants further research and clarification.

## 5. Concussion

These discoveries of core regulatory molecules, including HIF1A, adenosine, TBK1, ATP, NRAS, and EIF2AK3, unveil crucial signaling pathways and regulatory networks implicated in MI pathogenesis, providing novel perspectives for comprehensive understanding of this disease. Further exploration of the functions and interactions of these core regulatory molecules holds promise in offering novel targets and strategies for the treatment of ischemic heart disease. Therefore, in-depth investigation into the roles of these core regulatory molecules in ischemic heart disease offers vital theoretical and practical foundations for future therapeutic interventions.

## Figures and Tables

**Table 1:** Mendelian Randomization Estimation of the Relationship between Mitochondrial DNA Copy Number and Myocardial Infarction, Coronary Artery Bypass Grafting.

exposure	outcome	method	OR	p value
Mitochondrial DNA copy number	myocardial infarction	MR Egger	0.99316(0.97812-1.00843)	0.38
Mitochondrial DNA copy number	myocardial infarction	Weighted median	0.99233(0.98586-0.99883)	0.02
Mitochondrial DNA copy number	myocardial infarction	Inverse variance weighted	0.99372(0.98785-0.99961)	0.03
Mitochondrial DNA copy number	myocardial infarction	Simple mode	0.99189(0.97910-1.00485)	0.22
Mitochondrial DNA copy number	myocardial infarction	Weighted mode	0.99340(0.98441-1.00247)	0.16
Coronary artery bypass grafting	Mitochondrial DNA copy number	MR Egger	0.94940(0.91719-0.98272)	0.005
Coronary artery bypass grafting	Mitochondrial DNA copy number	Weighted median	0.98203(0.96628-0.99805)	0.028
Coronary artery bypass grafting	Mitochondrial DNA copy number	Inverse variance weighted	0.97672(0.95954-0.99421)	0.009
Coronary artery bypass grafting	Mitochondrial DNA copy number	Simple mode	0.99955(0.96554-1.03477)	0.98
Coronary artery bypass grafting	Mitochondrial DNA copy number	Weighted mode	0.98792(0.96641-1.00991)	0.283

**Table 2:** Heterogeneity Test between Mitochondrial DNA Copy Number and Myocardial Infarction, Coronary Artery Bypass Grafting.

outcome	exposure	method	Q	Q_df	Q_pval
Mitochondrial DNA copy number	Coronary artery bypass grafting	MR Egger	237.6645	60	5.47E-23
Mitochondrial DNA copy number	Coronary artery bypass grafting	Inverse variance weighted	251.4736	61	5.74E-25
myocardial infarction	Mitochondrial DNA copy number	MR Egger	94.92871	50	0.000130747
myocardial infarction	Mitochondrial DNA copy number	Inverse variance weighted	94.94027	51	0.000184057

**Table 3:** Horizontal Pleiotropy Test between Mitochondrial DNA Copy Number and Myocardial Infarction, Coronary Artery Bypass Grafting

outcome	exposure	egger_intercept	se	pval
Mitochondrial DNA copy number	Coronary artery bypass grafting	0.002487964	0.001333	0.066771
myocardial infarction	Mitochondrial DNA copy number	1.38E-05	0.000177	0.93811
